# DHHC5-mediated palmitoylation of S1P receptor subtype 1 determines G-protein coupling

**DOI:** 10.1038/s41598-017-16457-4

**Published:** 2017-11-29

**Authors:** Shaymaa Mohamed Mohamed Badawy, Taro Okada, Taketoshi Kajimoto, Takeshi Ijuin, Shun-ichi Nakamura

**Affiliations:** Division of Biochemistry, Department of Biochemistry and Molecular Biology, Kobe University Graduate School of Medicine, Kobe, 650-0017 Japan

## Abstract

Sphingosine 1-phosphate (S1P) is a pleiotropic lipid mediator involved in the regulation of immune cell trafficking and vascular permeability acting mainly through G-protein-coupled S1P receptors (S1PRs). However, mechanism underlying how S1PRs are coupled with G-proteins remains unknown. Here we have uncovered that palmitoylation of a prototypical subtype S1P_1_R is prerequisite for subsequent inhibitory G-protein (Gi) coupling. We have identified DHHC5 as an enzyme for palmitoylation of S1P_1_R. Under basal conditions, S1P_1_R was functionally associated with DHHC5 in the plasma membranes (PM) and was fully palmitoylated, enabling Gi coupling. Upon stimulation, the receptor underwent internalisation leaving DHHC5 in PM, resulting in depalmitoylation of S1P_1_R. We also revealed that while physiological agonist S1P-induced endocytosed S1P_1_R readily recycled back to PM, pharmacological FTY720-P-induced endocytosed S1P_1_R-positive vesicles became associated with DHHC5 in the later phase, persistently transmitting Gi signals there. This indicates that FTY720-P switches off the S1P signal in PM, while switching on its signal continuously inside the cells. We propose that DHHC5-mediated palmitoylation of S1P_1_R determines Gi coupling and its signalling in a spatio/temporal manner.

## Introduction

GTP-binding protein (G-protein)-coupled receptors (GPCRs) constitute one of the largest protein families in the human genome and transmit a wide variety of signals including odors, light, hormones and neurotransmitters, to control vital functions. GPCRs having common structural features, *i.e*., heptahelical transmembrane spans, are known to be regulated by diverse posttranslational modifications such as phosphorylation^1^ and palmitoylation^2^.

Protein palmitoylation is the attachment of the 16-carbon saturated fatty acid palmitate to cysteine residues on proteins through thioester linkages, and plays a role in the regulation of localisation, trafficking and function of a variety of proteins including GPCRs^2^. Rhodopsin was the first GPCR for which palmitoylation was demonstrated^3^. Subsequently a number of GPCRs such as the β2-adrenergic^4^, the α2-adrenergic receptor^5^, the D1 dopamine receptor^6^, the serotonin 5HT1B receptor^7^, the LH/hCG receptor^8^, and the glutamate mGluR4^9^ have been shown to be palmitoylated.

During the search for S-acyltransferases using Saccharomyces cerevisiae, Erf2 and Erf4 were identified as essential proteins for S-palmitoylation of Ras2^10^. Independently, Akr1 was shown to be an enzyme for S-palmitoylation of the yeast casein kinase Yck2^11^. These initial studies in yeast have lead to the discovery of a family of 23 mammalian DHHC (Asp-His-His-Cys) proteins as S-acyltransferases^12^.

Sphingosine-1-phosphate (S1P), a phosphorylated product of sphingosine catalysed by sphingosine kinase (SphK) has emerged as a potent lipid mediator with diverse effects on multiple biological processes^13,14^. S1P exerts its actions mainly through binding to a family of G-protein-coupled S1P receptors, termed S1P_1–5_ receptors (S1P_1–5_R)^15^ triggering diverse cellular processes, including cell angiogenesis, cardiac development, immunity, cell motility, neurotransmitter release and multivesicular endosome maturation^16–19^.

It has previously been shown that S1P_1_R is palmitoylated at three Cys residues in the cytoplasmic tail of the receptor^20^. However, the functional role of the receptor palmitoylation remains unknown in detail. In the present studies we have identified DHHC5 as an enzyme for palmitoylation of S1P_1_R. We have shown evidence that palmitoylation of the S1P_1_R is critical for the receptor coupling with Gi and that the extent of palmitoylation is dynamically regulated during receptor internalisation after activation. Physiological relevance of the association of DHHC5 with the S1P_1_R during vesicular trafficking is also discussed.

## Results

### Identification of DHHC5 as a palmitoyl acyltransferase for S1P_1_R

**Figure 1:**
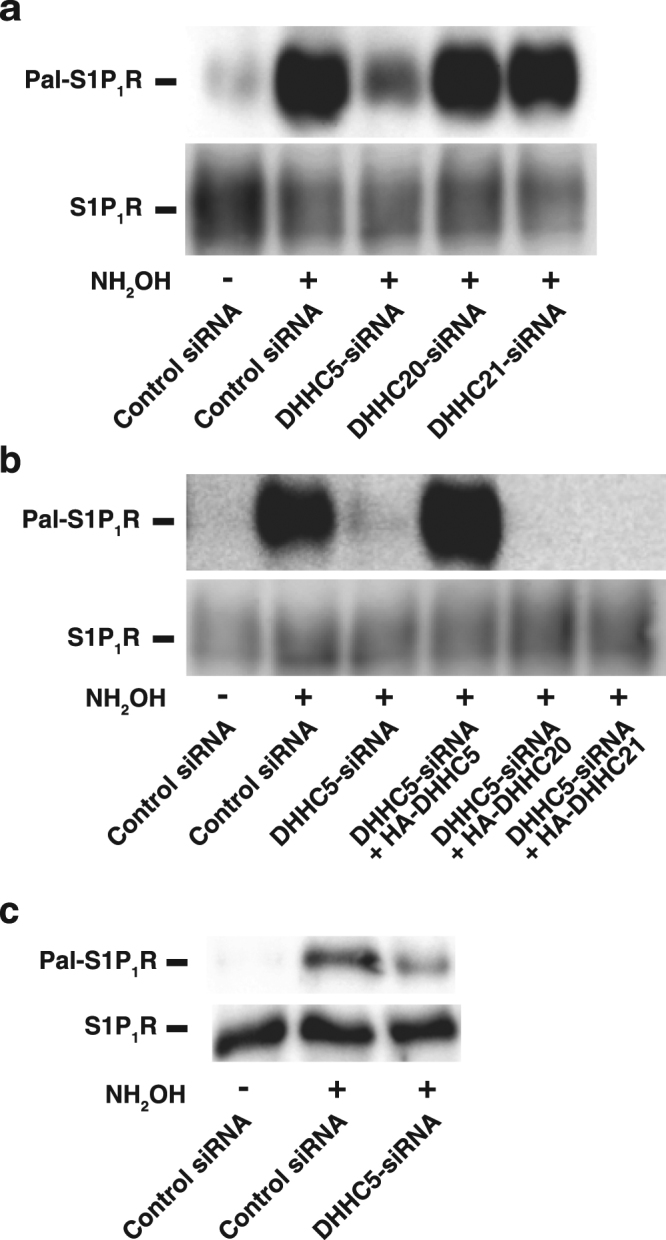
Identification of DHHC5 as an enzyme which palmitoylates S1P_1_R. (**a**) SH-SY5Y cells transiently transfected with a vector encoding S1P_1_R-FLAG together with control, DHHC5-, DHHC20- or DHHC21-siRNA were cultured for 72 hr. After cell lysis, immunoprecipitated S1P_1_R-FLAG was analysed for palmitoylation by ABE assay. NH_2_OH, hydroxylamine. Full-length images are presented in Supplementary Figure 6a. (**b**) SH-SY5Y cells transiently transfected with vectors encoding control (empty), S1P_1_R-FLAG, siRNA-resistant DHHC5, DHHC20, or DHHC21 together with control or DHHC5-siRNA were cultured for 72 hr. Palmitoylation of S1P_1_R was analysed as in (**a**). Full-length images are presented in Supplementary Figure 6b. (**c**) SH-SY5Y cells (1 × 10^7^ cells) transfected with control or DHHC5-siRNA were immunoprecipitated by an antibody against S1P_1_R followed by ABE assay as in (**a**). Full-length images are presented in Supplementary Figure 6c.

To identify an enzyme that palmitoylates S1P_1_R, the expression of several DHHC enzymes was down-regulated by a gene-silencing technique and the extent of palmitoylation of S1P_1_R was compared with a control siRNA treatment. Since S1P_1_R is localised predominantly at PM in SH-SY5Y cells as previously reported^21^ we reasoned that DHHC5, DHHC20 and DHHC21, which are known to show plasma membrane localisation^22^, may be potential candidates for the enzymes for S1P_1_R. The hydroxylamine-sensitive cleave of palmitate from proteins and exchange it with biotin (acyl-biotin exchange, ABE) method shows that S1P_1_R is a palmitoylated protein (Fig. 1a, control siRNA), as reported previously^20^. A condition without hydroxylamine serves as a negative control. It is noteworthy that down-regulation of DHHC5 by a specific siRNA treatment resulted in a robust reduction of palmitoylation of the receptor. Knockdown of DHHC20 or DHHC21 had little effect on the palmitoylation of the receptor (Fig. 1a, DHHC20-siRNA, DHHC21-siRNA). These results suggest that DHHC5 is a potential palmitoyl transferase for S1P_1_R. To further validate this notion, a rescue experiment was conducted next. Under DHHC5-downregulated conditions in a specific siRNA treatment, the expression of siRNA-resistant DHHC5 completely rescued the palmitoylation to that of control siRNA treatment, whereas DHHC20 or DHHC21 expression did not (Fig. 1b). The expression levels of DHHC20 and DHHC21 were comparable with that of siRNA-resistant DHHC5 (Supplementary Fig. 1). It is noteworthy that not only expressed S1P_1_R but the endogenous protein was also palmitoylated by DHHC5 since DHHC5 knockdown caused a clear reduction of palmitoylation of the endogenous S1P_1_R (Fig. 1c), suggesting a physiological relevance of this phenomenon.

### Association of S1P_1_R with DHHC5 under basal conditions

**Figure 2:**
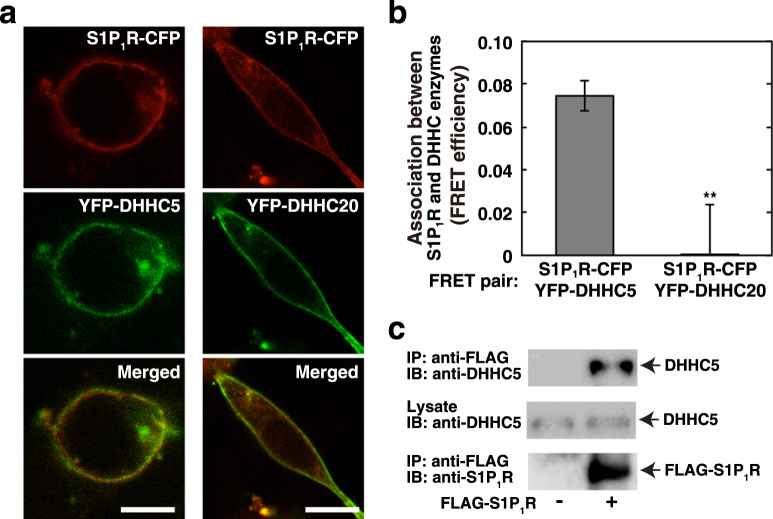
Association of DHHC5 with S1P_1_R. (**a**) SH-SY5Y cells transiently expressing S1P_1_R-CFP and either YFP-DHHC5 or YFP-DHHC20 were fixed and observed for each fluorescence by confocal laser scanning microscope. (**b**) Cells expressing various proteins as in (**a**) were fixed for FRET efficiencies. Values represent means ± s.e.m. of 3 independent experiments carried out in triplicate. Statistical significance was analysed by Student’s t-test (**P < 0.01 versus DHHC5-expressing cells). (**c**) Cells (2 × 10^7^ cells) expressing FLAG-S1P_1_R were lysed and immunoprecipitated by FLAG-antibody. The immunoprecipitates or lysates were subjected to immunoblot analysis using an anti-DHHC5 or an anti-S1P_1_R antibody. Representative images form 3 independent experiments are shown. Full-length images are presented in Supplementary Figure 7.

To investigate whether DHHC5 is a physiologically relevant enzyme for S1P_1_R palmitoylation, association of S1P_1_R with DHHC5 was studied next. When YFP-fused DHHC enzymes were expressed in the cells, both DHHC5 and DHHC20 were mainly localised in PM together with S1P_1_R (Fig. 2a). Then, the ability of these DHHC enzymes to interact with S1P_1_R was assessed by a FRET-based protein-protein interaction assay. Importantly, only DHHC5 showed close association with the receptor using S1P_1_R-CYP and each YFP-DHHC enzyme as a FRET pair (Fig. 2b). Furthermore, exogenously expressed FLAG-S1P_1_R was associated with endogenous DHHC5 as assessed by pull-down assay (Fig. 2c). This result suggests that S1P_1_R and DHHC5 are closely associated under basal conditions.

### Ligand-induced internalisation of S1P_1_R leaving DHHC5 at PM

**Figure 3:**
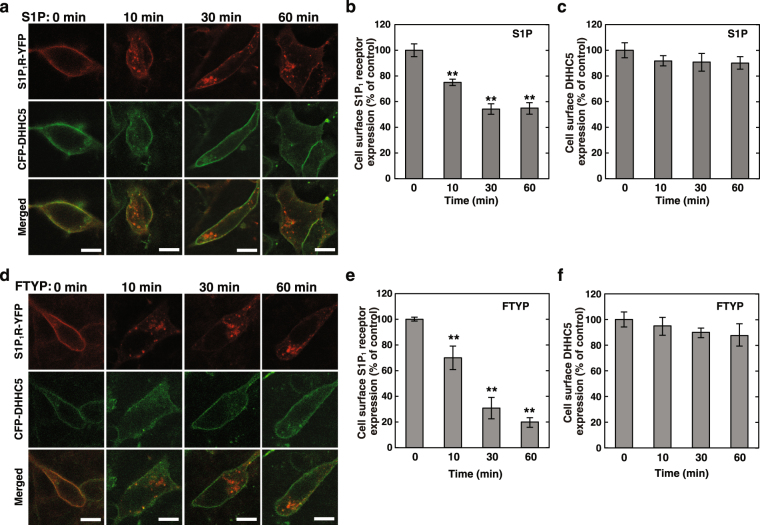
Ligand-induced internalization of S1P_1_R. (**a**,**d**) SH-SY5Y cells transiently expressing both S1P_1_R-YFP and CFP-DHHC5 were stimulated either with 100 nM S1P (**a**) or with 10 nM FTY720-P (**d**) for various time periods as specified. After fixation of the cells, the fluorescence of each protein was analysed by confocal microscopy. One of the representative results from three independent experiments is shown. (**b**,**c**,**e**,**f**) Fluorescence intensity of S1P_1_R-YFP (**b**,**e**) and CFP-DHHC5 (**c**,**f**) at each time point stimulated by S1P (**b**,**c**) or by FTY720-P (**e**,**f**) obtained in (**a**,**d**) were quantitated and compared with time point 0 (control). Values represent means ± s.e.m. of 3 independent experiments carried out in tripricate. Statistical significance was analysed by Student’s t-test (n = 40; **P < 0.01 versus control (0 min)).

To address whether the interaction of S1P_1_R with DHHC5 is tight enough for them to behave together during agonist-induced receptor internalisation or is controlled separately, the behaviour of both proteins was analysed after stimulation of cells by a physiological agonist S1P or a pharmacological one, phosphorylated-form of FTY720 (FTY720-P), which is clinically used as an immunosuppressant for multiple sclerosis and that acts through S1PRs^23^. Upon stimulation of cells with S1P, S1P_1_R underwent internalisation (Fig. 3a), being a plateau in 30 min with about half of the receptor internalised (Fig. 3b). Importantly, most of the DHHC5 remained in PM during the time course tested (Fig. 3c). FTY720-P caused a robust internalisation of the receptor, with 80% of the receptor internalised in 60 min (Fig. 3d,e). Similar to S1P, the majority of DHHC5 remained in PM (Fig. 3f). These results indicate that upon stimulation of the receptor, S1P_1_R behaves differently from DHHC5.

### Ligand-induced depalmitoylation of S1P_1_R

**Figure 4:**
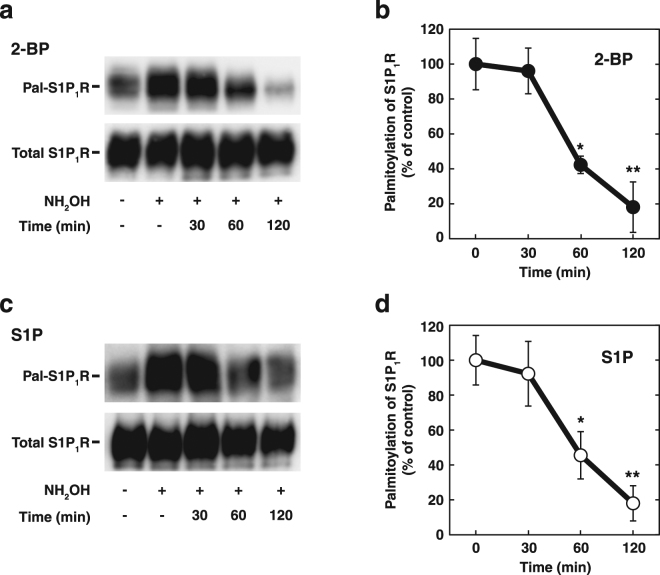
S1P-induced depalmitoylation of S1P_1_R. (**a**) SH-SY5Y cells transiently expressing S1P_1_R-FLAG were incubated with 150 µM 2-bromo-palmitate (2-BP) for the indicated time periods. After cell lysis, immunoprecipitated S1P_1_R-FLAG was analysed for palmitoylation by ABE assay. One of the representative results from three independent experiments is shown. Full-length images are presented in Supplementary Figure 8a. (**b**) The intensity of immunoreactive bands corresponding to palmitoylated S1P_1_R (Pal-S1P_1_R) at each time point obtained in (**a**) was quantitated. Values represent means ± s.e.m. of 3 independent experiments carried out in triplicate. Statistical significance was analysed by Student’s t-test (n = 9; **P < 0.01, *P < 0.05 versus control (0 min)). (**c**) SH-SY5Y cells transiently expressing S1P_1_R-FLAG were stimulated with 100 nM S1P for the indicated time periods. After cell lysis, immunoprecipitated S1P_1_R-FLAG was analysed for palmitoylation by ABE assay. One of the representative results from three independent experiments is shown. Full-length images are presented in Supplementary Figure 8b. (**d**) The intensity of immunoreactive bands corresponding to palmitoylated S1P_1_R at each time point obtained in (**c**) was quantitated. Values represent means ± s.e.m. of 3 independent experiments carried out in triplicate. Statistical significance was analysed by Student’s t-test (n = 9; **P < 0.01, *P < 0.05 versus control (0 min)).

Provided that palmitoylation and depalmitoylation are readily inter-convertible, it may be reasonable to assume that ligand-induced dissociation of S1P_1_R from DHHC5 (Fig. 3) may result in a depalmitoylation of the receptor. The next experiments were performed to compare the time course of depalmitoylation of the S1P_1_R induced by S1P and 2-bromo-palmitate (2-BP), a non-metabolisable palmitate analogue that blocks palmitate incorporation into proteins^24^. Treatment of cells with 2-BP resulted in a time-dependent decrease in palmitoylation of S1P_1_R (Fig. 4a,b). Two-hour incubation with the inhibitor caused almost unpalmitoylated level of the receptor (Fig. 4a, compare with no hydroxylamine). Notably, upon cell stimulation by S1P there was a reduction in the extent of S1P_1_R palmitoylation in a time course similar to 2-BP treatment (Fig. 4c,d). These results suggest that although depalmitoylation of the S1P_1_R is catalysed by the depalmitoylation enzyme, acyl-protein thioesterases (APT), the dissociation of the receptor from DHHC5 may be an important determinant of depalmitoylation of this protein.

### Essential role of S1P_1_R palmitoylation in receptor/G-protein coupling

**Figure 5:**
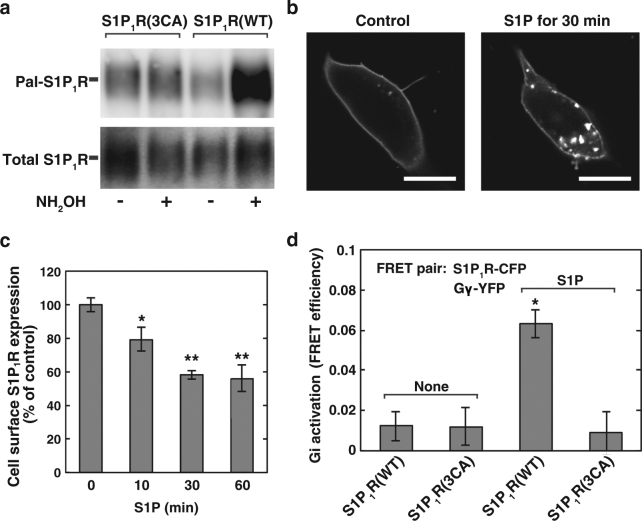
Palmitoylation-deficient mutant S1P_1_R(3CA) was unable to couple with Gi. (**a**) SH-SY5Y cells transiently expressing wild-type S1P_1_R-FLAG or S1P_1_R(3CA)-FLAG were analysed for palmitoylation by ABE assay. One of the representative results from three independent experiments is shown. Full-length images are presented in Supplementary Figure 9. (**b**) SH-SY5Y cells transiently expressing S1P_1_R(3CA)-CFP were stimulated with or without 100 nM S1P for 30 min and fixed with paraformaldehyde. The localisation of S1P_1_R(3CA)-CFP was analysed by confocal microscopy. Bars, 10 µm. One of the representative results from three independent experiments is shown. (**c**) SH-SY5Y cells transiently expressing S1P_1_R(3CA)-CFP were stimulated with 100 nM S1P for various time periods as specified. After fixation of the cells, the fluorescence of each protein was analysed by confocal microscopy. Fluorescence intensity at each time point was quantitated and compared with time point 0 (control). Values represent means ± s.e.m. of 3 independent experiments carried out in triplicate. Statistical significance was analysed by Student’s t-test (n = 9; **P < 0.01, *P < 0.05 versus control (0 min)). (**d**) SH-SY5Y cells transiently expressing wild-type S1P_1_R-CFP or S1P_1_R(3CA)-CFP together with Gβ and Gγ-YFP were stimulated with or without 100 nM S1P for 2 min, fixed and analysed for FRET efficiencies. Values represent means ± s.e.m. (n ≥ 50). Statistical significance was analysed by Student’s t-test (*P < 0.05 versus wild-type S1P_1_R with S1P).

To assess the functional role of palmitoylation of the receptor, we analysed a palmitoylation-deficient mutant protein. It has previously been reported that S1P_1_R is palmitoylated on three Cys residues in the C-terminal tail and a mutant (3CA), where all these three Cys residues were mutated to Ala, was shown to be palmitoylation-incompetent^20^. In fact, the 3CA mutant showed no palmitoylation as judged by ABE assay (Fig. 5a). When S1P_1_R(3CA) was expressed in the cells this mutant predominantly localised in PM under basal conditions (Fig. 5b, control) and showed both PM and internal vesicle localisation upon stimulation by S1P (Fig. 5b, S1P for 30 min). The pattern of agonist-dependent internalisation of the mutant was indistinguishable from that of the wild type (Figs 3b and 5c). To test the ability of the receptor to couple with Gi, we utilised FRET-based conformational studies to assess the receptor/G-protein coupling. Under basal conditions S1P_1_R is coupled with Giα in a heterotrimeric form and upon stimulation by S1P, Gβγ becomes associated with S1P_1_R after Gi subunit dissociation. Recently, using S1P_1_R-CFP and Gβγ-YFP as a FRET pair, agonist-induced S1P_1_R activation followed by Gi subunit dissociation was successfully demonstrated in our laboratory^19^. Using this system, wild-type S1P_1_R exhibited stimulation-dependent association with the Gβγ subunits (Fig. 5d, increased FRET efficiency), showing the receptor/Gi coupling. Importantly, when the 3CA mutant was expressed, this palmitoylation-deficient mutant failed to associate with the Gβγ subunits after S1P stimulation. These results suggest that palmitoylation of S1P_1_R is prerequisite for the receptor/Gi coupling.

### Essential role of palmitoylation of S1P_1_R in functional coupling of the receptor with Gi in an endogenous protein system

**Figure 6:**
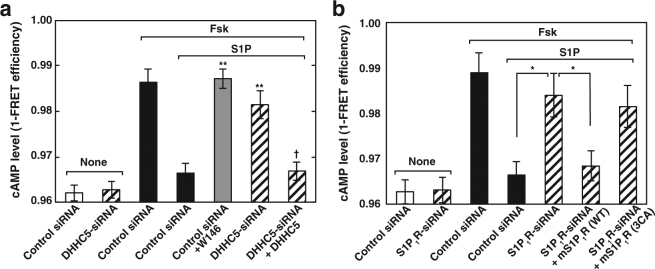
Correlation between DHHC5 association and S1P_1_R-coupling with Gi. (**a**) SH-SY5Y cells transiently transfected with a vector encoding cAMP biosensor Epac1-camps together with control or DHHC5-siRNA were cultured for 72 hr. In some experiments siRNA-resistant mCherry-DHHC5 was also expressed as specified. Cells were treated with 0.5 mM cAMP phosphodiesterase inhibitor, isobutylmethylxanthine, 20 µM forskolin in the presence or absence of 100 nM S1P or 10 µM W146 as indicated. Cells were fixed and analysed for FRET efficiencies. Values represent means ± s.e.m. of 3 independent experiments carried out in triplicate. Statistical significance was analysed by Student’s t-test (**P < 0.01 versus control siRNA treated with S1P; ^†^P < 0.05 versus DHHC5-siRNA treated with S1P). (**b**) SH-SY5Y cells transiently transfected with a vector encoding cAMP biosensor Epac1-camps, together with control or S1P_1_R-siRNA, were cultured for 72 hr. In some experiments wild-type mS1P_1_R-FLAG or mS1P_1_R(3CA)-FLAG was also expressed as specified. Cells were fixed and analysed for FRET efficiencies as in (**a**). Values represent means ± s.e.m. of 3 independent experiments carried out in triplicate. Statistical significance was analysed by Student’s t-test (*P < 0.05).

Although analysis with S1P_1_R(3CA) (Fig. 5) strongly supports the importance of the receptor palmitoylation in signalling, we cannot completely exclude the possibility that genetically induced mutation of the protein may have structural alterations and cause an artefact. For this concern, analysis using endogenous protein system is important. To further strengthen the notion that DHHC5-mediated palmitoylation of S1P_1_R is a prerequisite for subsequent receptor-mediated G-protein signalling, the effect of knockdown of DHHC5 using a gene-silencing technique on S1P_1_R-mediated Gi function was studied next. Since one of the canonical roles of Gi is to inhibit adenylate cyclase activity by Giα subunit^25^, S1P-induced inhibition of forskolin-stimulated adenylate cyclase activity was studied. As a positive control (control siRNA treatment), S1P caused a potent inhibition of cAMP production in an S1P_1_R-specific blocker W146-inhibitable manner (Fig. 6a). Remarkably, in DHHC5-knockdown cells S1P stimulation did not inhibit the adenylate cyclase activity, suggesting that Gi function is impaired in DHHC5-knockdown cells. Notably, the expression of siRNA-resistant DHHC5 completely rescued the impairment of the receptor-mediated Gi function. Furthermore, knockdown of S1P_1_R impaired S1P-induced inhibition of forskolin-stimulated adenylate cyclase activity as expected (Fig. 6b). This phenotype was rescued by the expression of mouse wild-type S1P_1_R but not by S1P_1_R(3CA). These results indicate that DHHC5-mediated palmitoylation of S1P_1_R is a prerequisite for functional coupling of the receptor with Gi.

### Regulation of S1P_1_R/Gi coupling through DHHC5-mediated palmitoylation in a spatio-temporal manner

**Figure 7:**
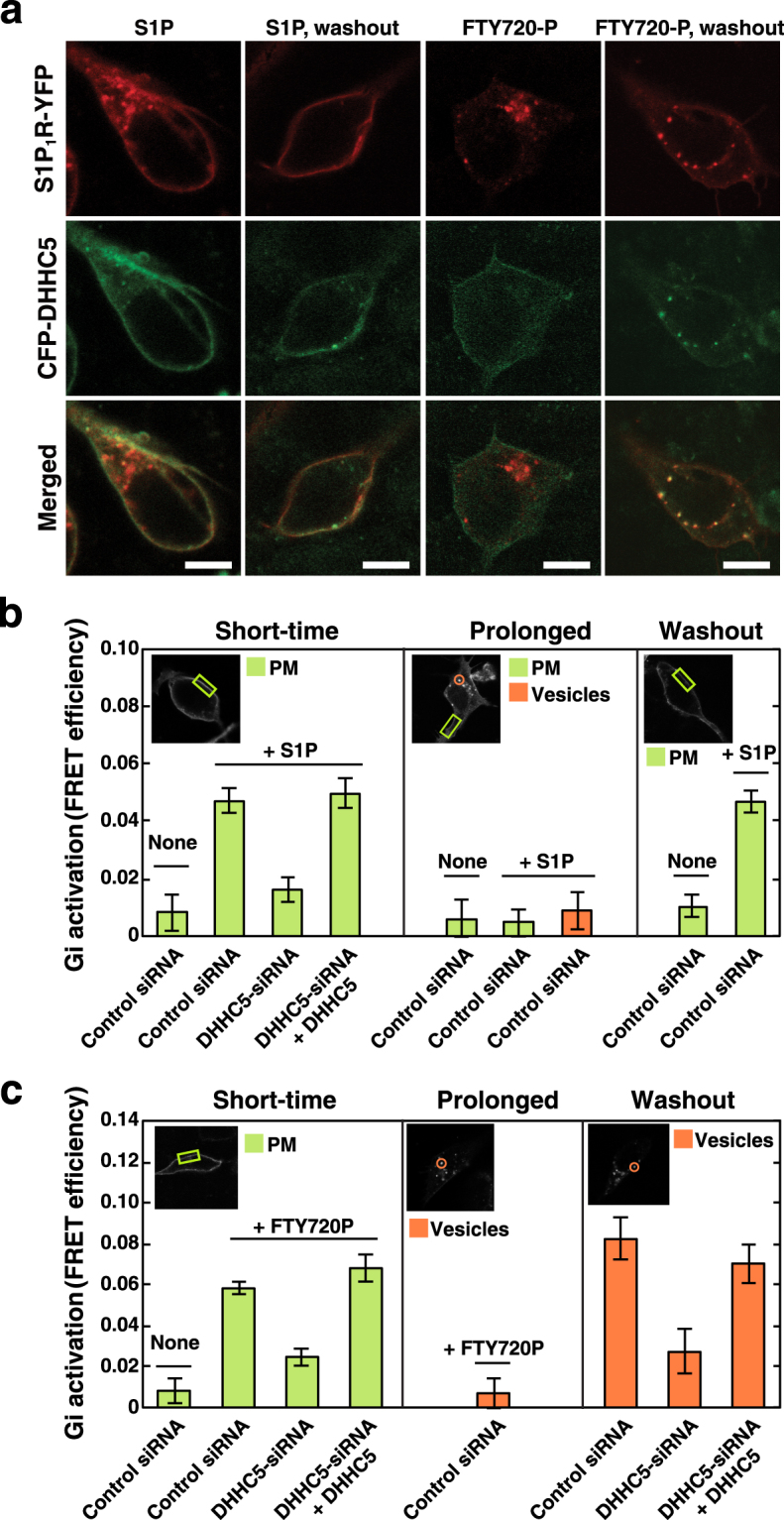
Essential role of DHHC5-mediated palmitoylation of S1P_1_R in functional coupling of the receptor with Gi. (**a**) SH-SY5Y cells transiently expressing both S1P_1_R-YFP and CFP-DHHC5 were stimulated either with 100 nM S1P or with 10 nM FTY720-P for 1 hr. In some experiments after 1-hr stimulation with each agonist, cells were washed and cultured for additional 5 hr in agonist-free media (washout). After fixation of the cells, the fluorescence of each protein was analysed by confocal microscopy. One of the representative results from three independent experiments is shown. (**b**) SH-SY5Y cells transiently transfected with a vector encoding S1P_1_R-CFP, Gβ and Gγ-YFP together with control or DHHC5-siRNA were cultured for 72 hr. In some experiments siRNA-resistant mCherry-DHHC5 was also expressed as specified. Cells were stimulated with 100 nM S1P for 2 min (short-time) or for 1 hr (prolonged) stimulation. In some experiments after 1-hr stimulation, cells were cultured for additional 5 hr in agonist-free media (washout) and then stimulated by 100 nM S1P for 2 min where indicated. Cells were fixed and analysed for FRET efficiencies. Values represent means ± s.e.m. (n ≥ 50). Insets show typical areas of PM (green boxes) or internal vesicles (red circles) used for FRET analysis in cells images detected by CFP signals. (**c**) SH-SY5Y cells transiently transfected with vectors and siRNAs as in (**b**) were stimulated with 10 nM FTY720-P for 2 min (short-time) or for 1 hr (prolonged) stimulation. In some experiments after 1-hr stimulation cells were cultured for additional 5 hr in agonist-free media (washout). Cells were fixed and analysed for FRET efficiencies. Values represent means ± s.e.m. (n ≥ 50). Note that under prolonged and washout conditions S1P_1_R-CFP signals in PM were too low to carry out FRET analysis, and this analysis in vesicles was conducted without any further agonist stimulation.

We have observed that agonist stimulation of the S1P_1_R causes internalisation of the receptor leaving DHHC5 in PM (Fig. 3), resulting in the depalmitoylation of the receptor (Fig. 4c,d). To understand the regulation of S1P_1_R-mediated cellular signalling with relevance to DHHC5-mediated palmitoylation of the receptor, behaviour of these proteins and Gi signalling after agonist stimulation were analysed in further details. Cells expressing both S1P_1_R-YFP and CFP-DHHC5 were treated with cycloheximide to inhibit *de novo* protein synthesis and were analysed for the cellular localisation of the two fluoro-proteins. Under basal conditions both proteins were colocalised in PM (Fig. 3a,d). Prolonged stimulation with S1P (1 hr) caused a prominent internalisation of S1P_1_R in contrast to the majority of DHHC5, which remained in PM (Figs 3a and 7a). Further cell culture for 5 hr in the absence of S1P after prolonged stimulation with this agonist resulted in almost all internalised S1P_1_R being recycled back to PM (Fig. 7a, see S1P, washout). In the case of prolonged FTY720-P stimulation, this pharmacological agonist induced a profound effect on the internalisation of the receptor (Figs 3d and 7a) while having a modest influence on DHHC5 localisation similarly to S1P (Figs 3d and 7a). Surprisingly, under the washout conditions after prolonged FTY720-P stimulation, internalised S1P_1_R persistently localised in the intracellular vesicles associated with DHHC5 (Fig. 7a, FTY720-P, washout). During the first 1 hr of washout periods, most of DHHC5 remained in PM and it was then internalised towards S1P_1_R-positive vesicles, reaching a maximum in 3 hr and then a plateau as shown in a time-lapse video recording (Supplementary Fig. 2).

To further evaluate the function of DHHC5-mediated palmitoylation of S1P_1_R in terms of Gi signalling, the receptor-mediated Gi activation (subunit dissociation) was monitored in various stages of the receptor after agonist stimulation. In control siRNA-treated cells short-time stimulation (2 min) with either S1P or FTY720-P caused Gi activation (FRET increase) (Fig. 7b,c). In this short time stimulation, internalised vesicles were not clearly detected (data not shown). Importantly, when DHHC5 was knocked down by siRNA, agonist-induced Gi activation was strongly inhibited, which was rescued by the expression of siRNA-resistant DHHC5 (Fig. 7b,c). In the prolonged stimulation (1 hr) with these agonists, the FRET efficiency was low in S1P_1_R-positive internal vesicles (Fig. 7b,c, vesicles), where majority of DHHC5 was not colocalised (Figs 3a,d and 7a). The FRET efficiency in PM was also low in the prolonged stimulation with S1P although DHHC5 was also in PM. Further analysis revealed that FRET values between S1P_1_R-CFP and YFP-DHHC5 in PM under basal conditions showed high, whereas after prolonged stimulation with S1P it was low in PM (Supplementary Fig. 3), suggesting that these proteins were physically distant in PM after prolonged stimulations. Under the washout conditions (further 5 hr-incubation without the agonist after the prolonged S1P stimulation), the recycled S1P_1_R in PM was competent to respond to S1P for Gi signalling (Fig. 7b, washout). Intriguingly, under washout conditions after the prolonged FTY720-P stimulation, Gi was constantly activated (high FRET) on S1P_1_R-positive vesicles (Fig. 7c, washout, control siRNA). Notably, this continuous activation of S1P_1_R/Gi was not observed in DHHC5-knockdown cells (see DHHC5-siRNA). Furthermore, DHHC5-siRNA-induced impairment of S1P signalling on these vesicles was completely rescued by the expression of siRNA-resistant DHHC5 (see DHHC5-siRNA + DHHC5).

Collectively, these results are suggestive of the notion that association of the S1P_1_R with DHHC5 at any compartments regulates palmitoylation of the receptor and has a pivotal role in the regulation of S1P_1_R-mediated Gi signalling.

## Discussion

We have identified DHHC5 as a protein palmitoyl transferase for S1P_1_R. Although there are 23 DHHCs so far reported in mammals, DHHC5 may be a sole enzyme for S1P_1_R since gene-targeted knockdown of the enzyme resulted in abolishment of the receptor palmitoylation, which was completely rescued by the expression of siRNA-resistant DHHC5 but not by other enzymes, DHHC20 and DHHC21 (Fig. 1), which are known to be localised in PM^22^. Palmitoylation of the S1P_1_R is shown to be necessary for coupling of the receptor with Gi since palmitoylation-defect mutant S1P_1_R(3CA) shows unresponsiveness to agonist-induced Gi subunit dissociation (Fig. 5d). A causal relationship between palmitoylation of the S1P_1_R and Gi-protein coupling was further substantiated by the effect of gene-targeted knockdown of DHHC5. Decreased palmitoylation of endogenous S1P_1_R in DHHC5-siRNA transfected cells has been shown to impair downstream Gi functions as judged by the inhibition of forskolin-stimulated adenylate cyclase activity, which is completely rescued by the expression of siRNA-resistant DHHC5 (Fig. 6).

As for palmitoylation/G-protein coupling, it has been shown that palmitoylation of the β2-adrenergic receptor plays an important role in receptor coupling with Gs. When the palmitoylation site of the β2-adrenergic receptor was mutated, the receptor failed to couple with Gs and resulted in defective activation of adenylate cyclase^4^. In contrast, among the two actions of rhodopsin, *i.e*., a photoreceptor and a chemical receptor for the diffusible agonist, all-trans-retinal, palmitoylation of rhodopsin has been shown to have little influence on its light-induced signalling^26^, but for the all-trans-retinal binding to opsin, palmitoylation of the protein plays an important role in the chromophore binding to opsin and its activation^27^. Palmitoylation of GPCRs may control fine tuning of downstream signalling depending on the receptor types. Palmitoylation of the S1P_1_R may have little influence on the protein localisation since the S1P_1_R(3CA) shows plasma membrane localisation (Fig. 5b) similar to the wild-type receptor. Likewise, the S1P_1_R(3CA) shows an agonist-induced internalisation of the receptor similar to the wild type (Figs 3c and 5c) in contrast to the striking difference of Gi coupling to the receptor (Fig. 5d), suggesting that S1P_1_R may employ distinct signalling molecules for diverse downstream events, *e.g*., internalisation and Gi signalling. To support this notion it has previously been reported that disruption of lipid raft by the cholesterol-depleting agent methyl-β-cyclodextrin caused impairment of G-protein effector signalling but not α_1a_-adrenergic receptor internalisation^28^. Further studies are necessary to clarify the molecular mechanism underlying the association of S1P_1_R with DHHC5 presumably in the membrane rafts.

A previous report suggests that palmitoylation of S1P_1_R increased upon agonist stimulation as measured by a radioisotope-labelling technique^20^. In contrast, our present results show an S1P-induced decrease in the palmitoylation of S1P_1_R as estimated by the ABE method (Fig. 4c,d). The discrepancy between the two results may come from differing experimental methods for detecting palmitoylation. With a pulse-labelling technique radioactive palmitate tends to be incorporated preferentially into increased-turnover molecules but not reflect a true kinetic property of palmitoylation. Similar observations were made in the β2-adrenergic receptor. Short-term incubation of the Sf9 cells expressing β2-adrenergic receptor with the agonist isoproterenol led to an increase in the incorporation of tritiated palmitate into the receptor^29^. This increase was more obvious with 2 hr pulse labelling with tritiated palmitate than 4 hr labelling. Subsequent analysis revealed that isoproterenol stimulation of the β2-adrenergic receptor resulted in an increase in the turnover rate of palmitoylation into the receptor and a sustained agonist stimulation caused a decrease in receptor palmitoylation^30^.

In the present studies prolonged incubation (1 hr) with either S1P or FTY720-P made S1P_1_R uncoupled from Gi (Fig. 7b,c, prolonged) in keeping with dissociation of the receptor from DHHC5 (Figs 3 and 7a). These DHHC5-negative and S1P_1_R-positive vesicles were colocalised with an early endosome marker, EEA1 (Supplementary Fig. 4). During washout processes after prolonged S1P stimulation almost all the internalised S1P_1_R was recycled back to PM. Unexpectedly, subsequent culture for 5 hr without the agonist after prolonged FTY720-P stimulation resulted in the internalisation of DHHC5 (Fig. 7a, FTY720-P washout, Supplementary Fig. 2) and consequently colocalised with S1P_1_R-positive internal vesicles (Fig. 7a), continuously sending Gi signals (Figs 6 and 7c, washout). DHHC5 and S1P_1_R-positive internal vesicles were each stained with both an early endosome marker, EEA1 and a late endosome marker CD63, suggesting hybrid endosomes (Supplementary Fig. 4). Some irregularly fused vesicles near the perinuclear areas were also stained with a trans Golgi marker TGN46 as previously suggested^31^. These results suggest that FTY720-P-induced internal vesicles undergo abnormal maturation processes, presumably keeping transmitting Gi signals, to multiple destinations.

Recently studies from our laboratory have shown that the S1P_1_R is continuously activated and transmitting Gi signals on multivesicular endosomes through S1P-mediated fashion, which is necessary for cargo sorting into exosomal intralumenal vesicles^19^. To support this phenomenon in terms of palmitoylation of the receptor, the S1P_1_R is associated with DHHC5 in constitutively active Rab5-positive endosomes where Gi subunits are dissociated (active) (Supplementary Fig. 5). Since DHHC5 internalisation is beyond the control of S1P (Fig. 3), it is still unclear how DHHC5 is targeted to Rab5-positive endosomes or FTY720-P induced internalised S1P_1_R-positive vesicles. It may plausible to assume some “insideout signallings” from internal vesicles to PM for the internalisation of DHHC5. Further studies are necessary to clarify the mechanism underlying DHHC5 trafficking and the activity control.

In conclusion, the present studies clearly show that DHHC5-mediated palmitoylation of the S1P_1_R plays a pivotal role in transmitting Gi signals in a spatio-temporal manner in the cells.

## Methods

### Antibodies and reagents

Anti-FLAG and anti-GFP antibodies were obtained from Cell Signaling Technology. Anti-EEA1 antibody and anti-CD63 antibody were obtained from BD Bioschiences; anti TGN46 antibody from Novus Biologicals; anti-S1P_1_ receptor rabbit polyclonal antibody from Santa Cruz Biotechnology; anti-ZDHHC5 rabbit polyclonal antibody from Proteintech Group. S1P was purchased from Enzo Life Sciences; PTX, forskolin and 2-bromo-palmitate from Wako Pure Chemical Industries; W146 from Cayman Chemical Company; hydroxylamine from Tokyo Chemical Industry; FTY720-P from Echelon Biosciences. Other reagents and chemicals were of analytical grade.

### Plasmids and mutations

Human DHHC5 (Accession number NM_015457), DHHC20 (Accession number NM_153251) and DHHC21 (Accession number NM_178566) were amplified by using 5′-TGTAGATCTATGCCCGCAGAGTCTGGAAAG-3′ and 5′-TATGTCGACTCACACCGAAATCTCATAGGTG-3′, 5′-AGCAGATCTATGGCGCCCTGGACGCTGTG-3′ and 5′-GATGTCGACTCATACACCTGATTTGACGATGC-3′ and 5′-AGAGTCGACATGGGTCTCCGGATTCACTTTG-3′ and 5′-AGAGGATCCTTAGACATGATTGGCAAAGTGG-3′, respectively, and cloned into pECFP-C1 (Clontech), pEYFP-C1 (Clontech), pmCherry-C1 (Clontech) and HA-tag-inserted pCMV5. Site-directed mutagenesis was performed using QuikChange protocol for siRNA-resistant DHHC5 silent mutant (5′-GCAGTGCCCATCTATAACGCGATCATGTTTCTCTTTGTG-3′ and its reverse complement.

Site-directed mutagenesis for mS1P_1_R(3CA) was performed using mutagenic synthetic oligonucleotides (5′-CATCCGGATCGTATCTGCCGCCAAAGCCCCCAACGGAGACTCTG-3′ and its reverse complement). S1P_1_ receptor-YFP was prepared as described previously^21^. mS1P_1_ receptor-CFP, Giα-CFP, Gβ and Gγ-YFP plasmid constructs were prepared as described previously^18^. FLAG-tagged Rab5(Q79L) was obtained as described previously^19^. A one-molecular FRET probe for detection of cAMP, Epac1-camps, was constructed as reported previously^32^. All the constructs were verified by sequencing.

### siRNA

For RNA interference following oligonucleotides (Japan Bio Services, Saitama, Japan) were used: Sense 5′-AAACAUAAUUGCAUUGUAGdAdT-3′ and antisense 5′-CUACAAUGCAAUUAUGUUUdCdT-3′ for human DHHC5; sense 5′-UCCAAAAAUAGUAAACACGdCdA-3′ and antisense 5′-CGUGUUUACUAUUUUUGGAdAdA-3′ for human DHHC20; sense 5′-UAAAACAAUAUUGUAUAACdCdA-3′ and antisense 5′-GUUAUACAAUAUUGUUUUAdAdT-3′ for human DHHC21.

SH-SY5Y cells were transfected with the siRNAs using Lipofectamine RNAiMAX according to the manufacturer’s instructions (Invitrogen, Carlsbad, CA, USA).

### Cell cultures and transfections

SH-SY5Y cells (American Type Culture Collection, CRL-2266) were maintained in DMEM/F-12 medium (Wako Pure Chemical Industries) containing 10% fetal bovine serum and 1% penicillin/streptomycin at 37 °C in 5% CO_2_. Cells were plated onto 35 mm glass-bottom culture dishes (MatTek) before transfection. Transient transfection was carried out using FuGENE HD (Promega). All experiments were performed 2 to 3 days after transfection.

### S1P_1_R and DHHC5 internalisation assay

SH-SY5Y cells transfected with S1P_1_R-YFP and CFP-DHHC5 were treated with 100 nM S1P or 10 nM FTY720-P for the indicated periods and fixed by 4% paraformaldehyde/PBS. The confocal microscope images of each cell were analysed by Image J software and percentage of signal intensity on the cell surface was calculated.

### Acceptor photobleaching

SH-SY5Y cells were transiently cotransfected with S1P_1_R-CFP, Gβ and Gγ-YFP^19^, with a donor/acceptor ratio of 1:1:1, with one-molecule cAMP probe, Epac1-camps^32^, with Giα-CFP, Gβ and Gγ-YFP^19^ or Giα-CFP and S1P_1_R-YFP. Two days after transfection, cells were treated with various reagents. Cells were then fixed and each area of interest was subjected to FRET analysis with acceptor photobleaching method using a LSM 510 META with a 63 x oil plan-apochromat objective. Following excitation at 458 or 514 nm, CFP emission with a 475–525-nm band-pass barrier filter or YFP emission with 530–600-nm band-pass barrier filter, respectively, was collected. An area of interest was selected for photobleaching of YFP. An automated acquisition protocol was then used, which recorded pre- and post-bleaching images using 458 nm excitation at 8% laser power to limit photobleaching, with a bleaching of the selected area with 100%, 514 nm laser power with 50 iterations (acceptor photobleaching). FRET was resolved as an increase in the CFP (donor) signal after photobleaching of YFP (acceptor). FRET efficiency (E) can be determined from the relative fluorescence intensity of the energy donor (CFP) before (Ipre) and after (Ipost) photobleaching of the energy acceptor (YFP): E = 1- (Ipre/Ipost).

### Acyl-biotin exchange (ABE) assay

An ABE assay was carried out as described with minor modifications^20^. SH-SY5Y cells transfected with FLAG-S1P_1_R were solubilised by lysis buffer (50 mM Tris-HCl (pH 7.4), 150 mM NaCl, 1 mM EDTA, 1% Triton X-100 and protease inhibitors) supplemented with 10 mM NEM and cleared by centrifugation at 15,000 x g for 5 min at 4 °C. The supernatant was incubated overnight with anti-FLAG tag antibody beads (Wako). The beads were washed twice with lysis buffer containing 10 mM NEM, and suspended in buffer B (50 mM Tris-HCl (pH 7.4) 150 mM NaCl, 5 mM EDTA, 1% Triton X-100 and 0.1% SDS) containing 50 mM NEM. After rotating the sample tube for 1 hr at room temperature, beads were washed twice with buffer B and once with 10 mM Tris-HCl (pH 7.4), then suspended in buffer C (10 mM Tris-HCl (pH 7.4) 0.2% Triton X-100, 150 mM NaCl and 0.2 mM EZ-Link Biotin-HPDP (Pierce)) containing 1 M hydroxylamine (pH 7.4) or 50 mM Tris-HCl (pH 7.4), and incubated for 1 hr at room temperature. Beads were washed twice with buffer B and once with 10 mM Tris-HCl (pH 7.4) and bound proteins were eluted with 80 µl of elution buffer (50 mM Tris-HCl (pH 7.4) 2% SDS and 5 mM EDTA). Samples were then diluted with 20 volumes of buffer D (50 mM Tris-HCl (pH 7.4) 150 mM NaCl, 5 mM EDTA and 1% Triton X-100) and incubated with streptavidin agarose (Solulink) overnight at 4 °C with rotation. Beads were washed twice with buffer B and once with 10 mM Tris-HCl (pH 7.4) and bound proteins were eluted with SDS sample buffer containing 10 mM DTT and subjected to immunoblotting with anti-S1P_1_R antibody (Santa Cruz).

### Statistical analysis

Results are expressed as means ± s.e.m. Data were analysed by t-test. P-values < 0.05 were considered significant.

## Electronic supplementary material

Supplementary Figures
