# Supplementary material for: DHHC5-mediated palmitoylation of S1P receptor subtype 1 determines G-protein coupling
Source: Sci Rep. 2017 Nov 29;7:16552. doi: 10.1038/s41598-017-16457-4 (PMC5707436; doi:10.1038/s41598-017-16457-4)
Supplement: Supplementary file 1 — Supplementary Figures [file 41598_2017_16457_MOESM1_ESM.pdf]

DHHC5-mediated palmitoylation of S1P receptor subtype 1 determines G-protein coupling

Shaymaa Mohamed Mohamed Badawy*, Taro Okada*, Taketoshi Kajimoto, Takeshi Ijuin & Shun-ichi Nakamura[1]

Division of Biochemistry, Department of Biochemistry and Molecular Biology, Kobe University Graduate School of Medicine, Kobe 650-0017, Japan.

[1]Corresponding author: snakamur@kobe-u.ac.jp

*These authors contributed equally to this work.

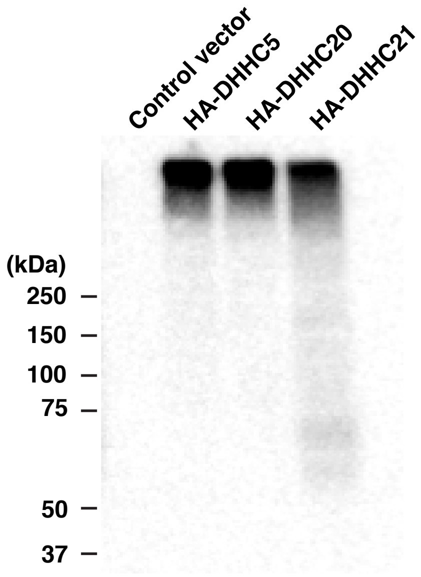

Supplementary Figure 1. Expression of siRNA-registant DHHC enzymes.

SH-SY5Y cells transiently transfected with vectors encoding siRNA-resistant HA-DHHC5, HA-DHHC20, or HA-DHHC21 were cultured for 72 hr. After cell lysis, the lysates were analysed by immunoblotting anti-HA antibody. Note that DHHC enzymes are highly self-aggregatable and subjected to temperature and reducing agents during preparation of the samples (Kokkola, T. et al. Somatostatin receptor 5 is palmitoylated by the interacting ZDHHC5 palmitoyltransferase. FEBS Lett. 585:2665-2670 (2011)). Therefore, expression of each protein was evaluated by the amount of high molecular weight-band compared with the control vector treatment.

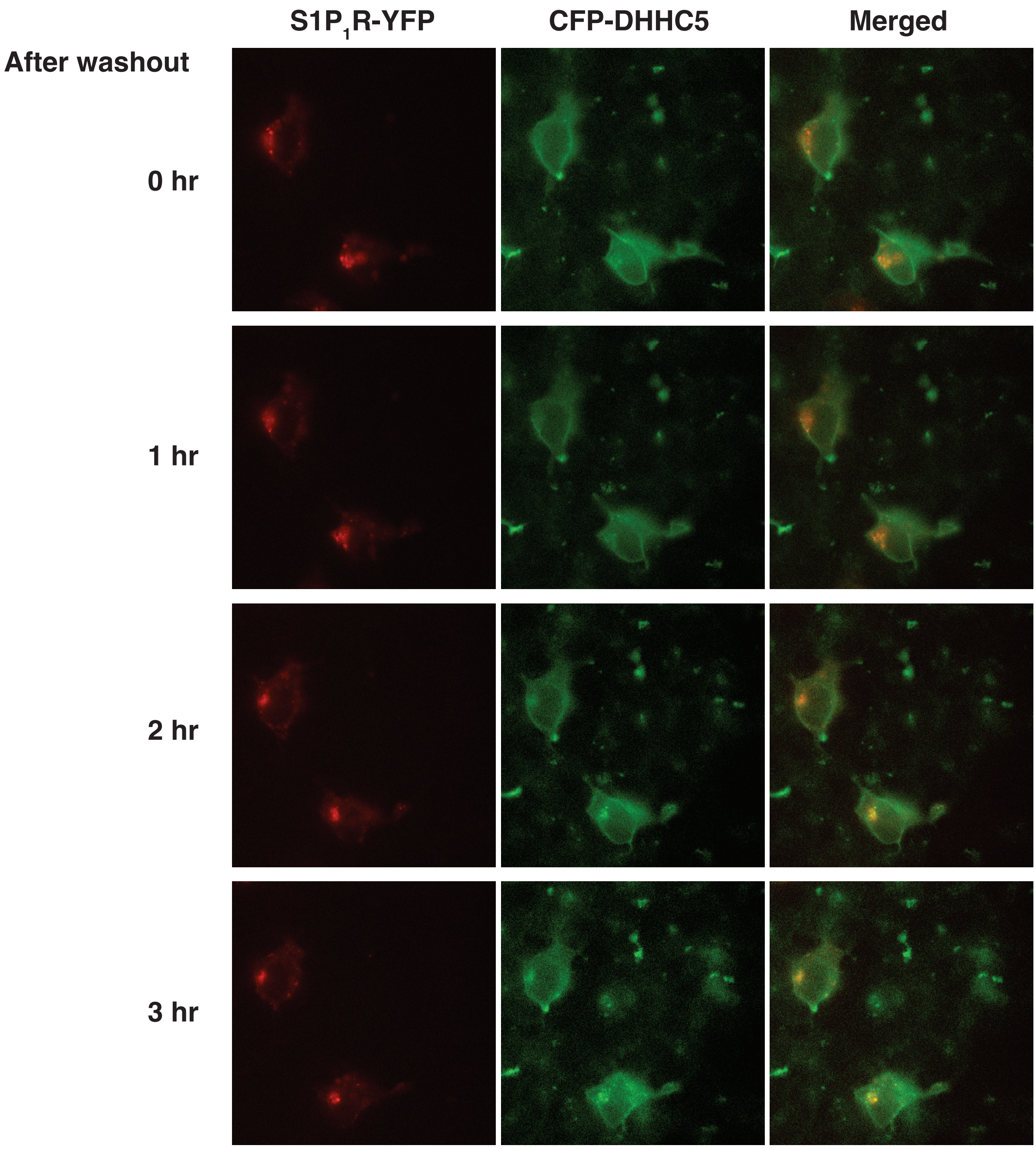

Supplementary Figure 2. Internalisation of DHHC5 during washout processes after prolonged (1 hr) treatment of FTY720-P.

SH-SY5Y cells transiently expressing both $S1P_1R$-CFP and DHHC5-YFP were stimulated with 10 nM FTY720-P for 1 hr. After cells were cultured in agonist-free media (washout), cells were sequentially monitord using a fluorescence microscope equipped with time-lapse recording system. Images were captured every hour for 3 hrs. One of the representative results from three independent experiments is shown.

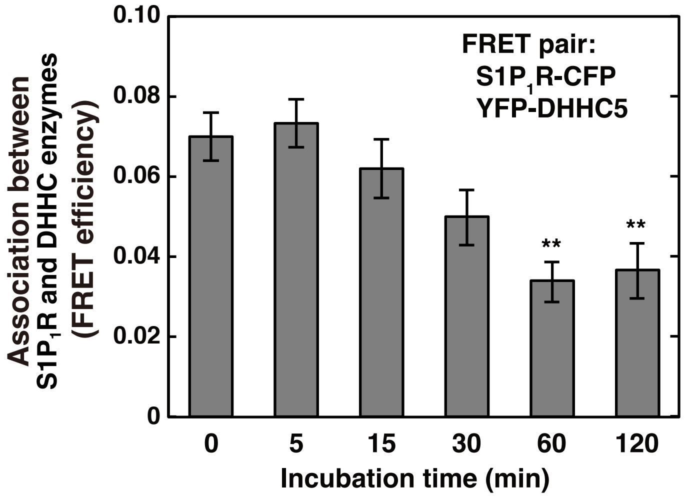

Supplementary Figure 3. S1P-induced dissociation of DHHC5 form S1P$_1$R in a time-dependent manner.

SH-SY5Y cells transiently expressing both S1P$_1$R-CFP and DHHC5-YFP were stimulated with 100 nM S1P for the indicated time periods. Cell were then fixed and analused for FRET efficiencies. Values represent means ± s.e.m. of 3 independent experiments carried out in triplicate. Statistical significance was analysed by Student' s t-test (**P < 0.01 versus 0 time).

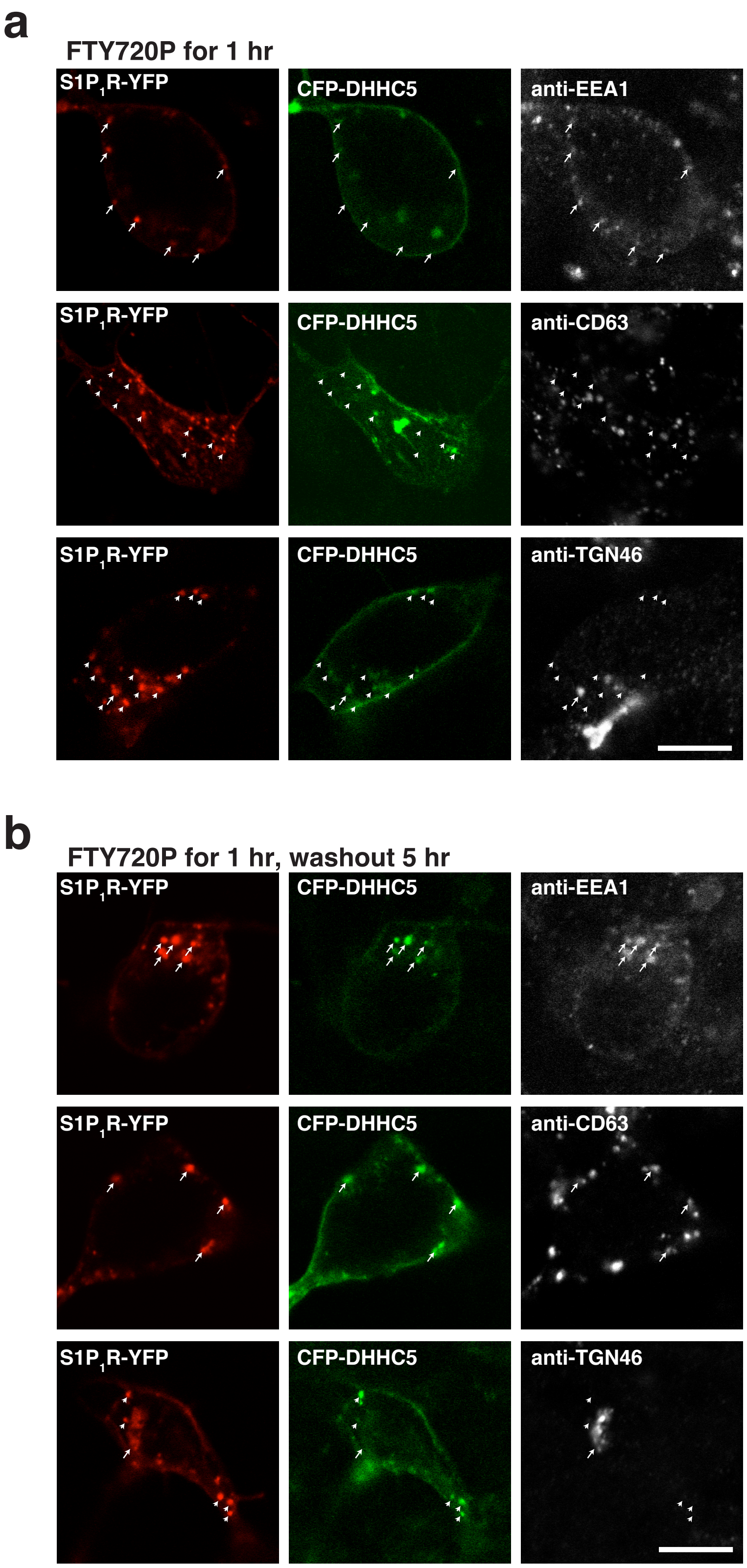

Supplementary Figure 4. Characterisation of FTY720-P-induced internalised vesicles with several vesicle markers.

(**a**) SH-SY5Y cells transiently expressing both S1P$_1$R-YFP and DHHC5-CFP were stimulated with 10 nM FTY720-P for 1 hr. After fixation, cells were immunostained with anti-EEA1, anti-CD63, or anti-TGN46 antibody and analysed by confocal microscopy. Scale bars, 10 μm.
(**b**) After prolonged incubation with FTY720-P as in (**a**), the culture media were changed with the ones washout agonist and cultured for additional 5 hrs. THe cells were treated as above and analysed by confocal microscopy. Scale bars, 10 μm.

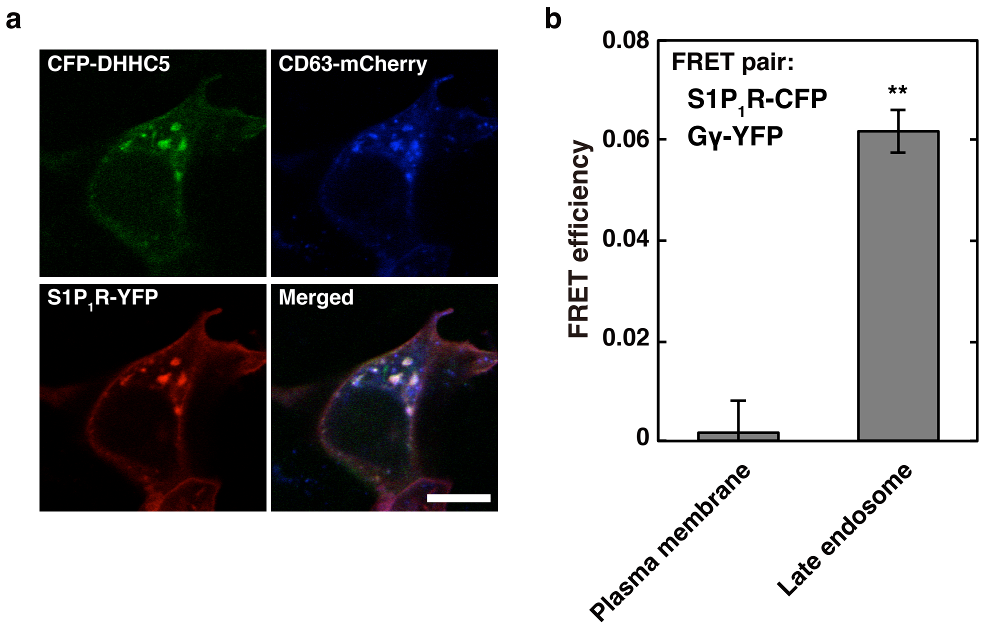

Supplementary Figure 5. S1P-induced dissociation of DHHC5 form S1P$_1$R in a time-dependent manner.

(**a**) SH-SY5Y cells transiently expressing both S1P$_1$R-YFP and CFP -DHHC5 were fixed and analysed for each fluorescence by confocal microscopy. One of the representative results from three independent experiments is shown.

(**b**) SH-SY5Y cells transiently transfected with a vector encoding S1P1R-CFP, Gβ, Gγ-YFP, Rab5 (Q79L)-DsRed and CD63-mCherry were cultured for 72 hr. After fixation, PM areas and CD63-positive enlarged vesicle areas were separately analysed for FRET efficiency using confocal microscopy. Values represent means ± s.e.m. of 3 independent experiments carried out in triplicate. Statistical significance was analysed by Student' s t-test (**P < 0.01 versus PM areas).

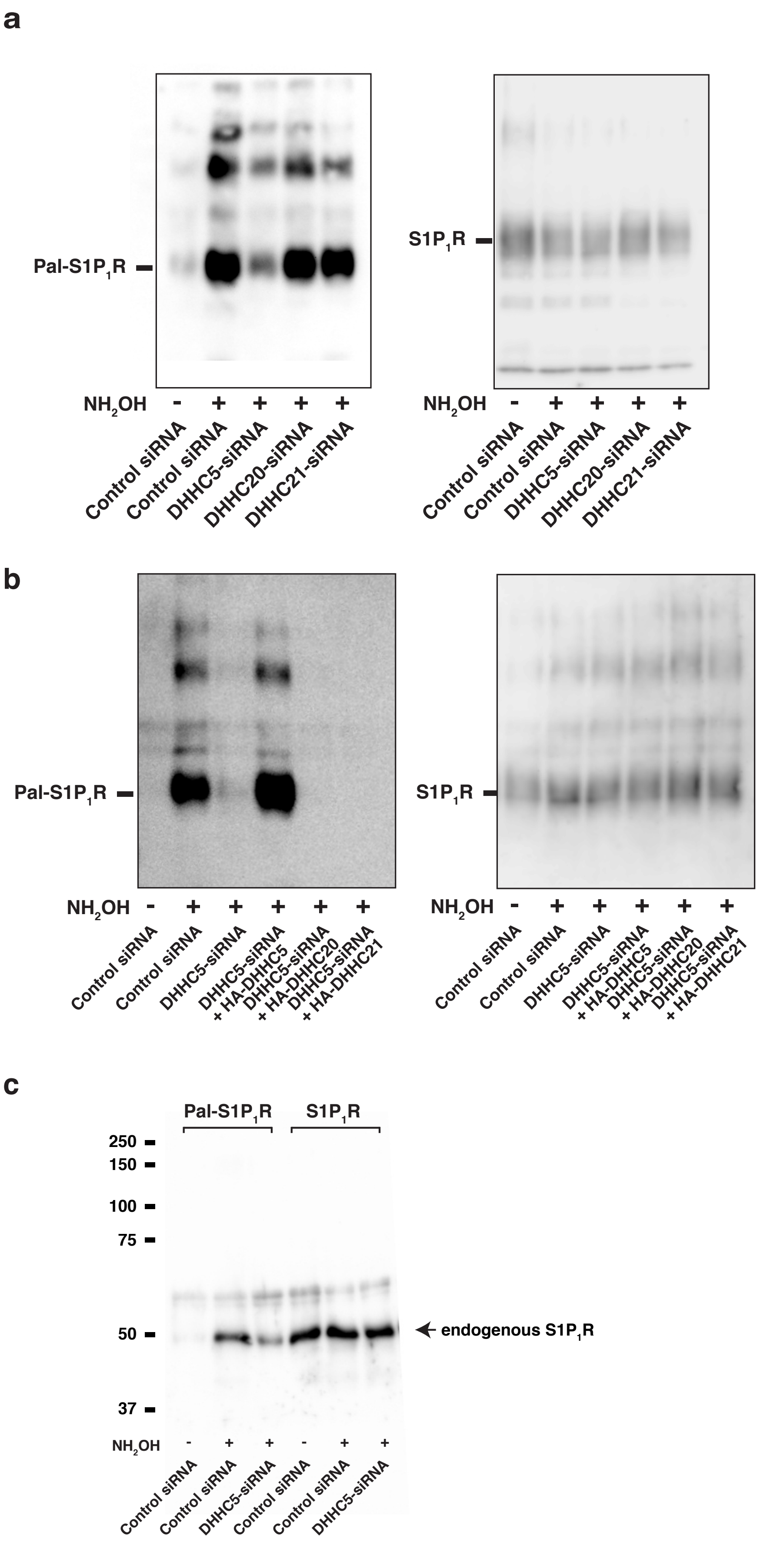

Supplementary Figure 6. Full-length image of western blot analysis in Figure 1.

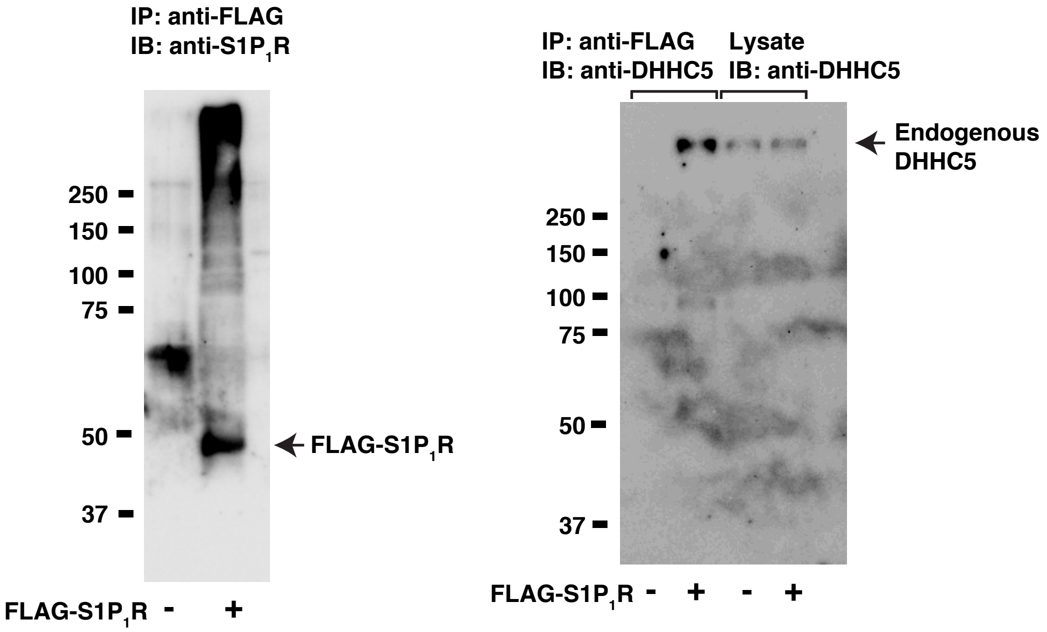

Supplementary Figure 7. Full-length image of western blot analysis in Figure 2.

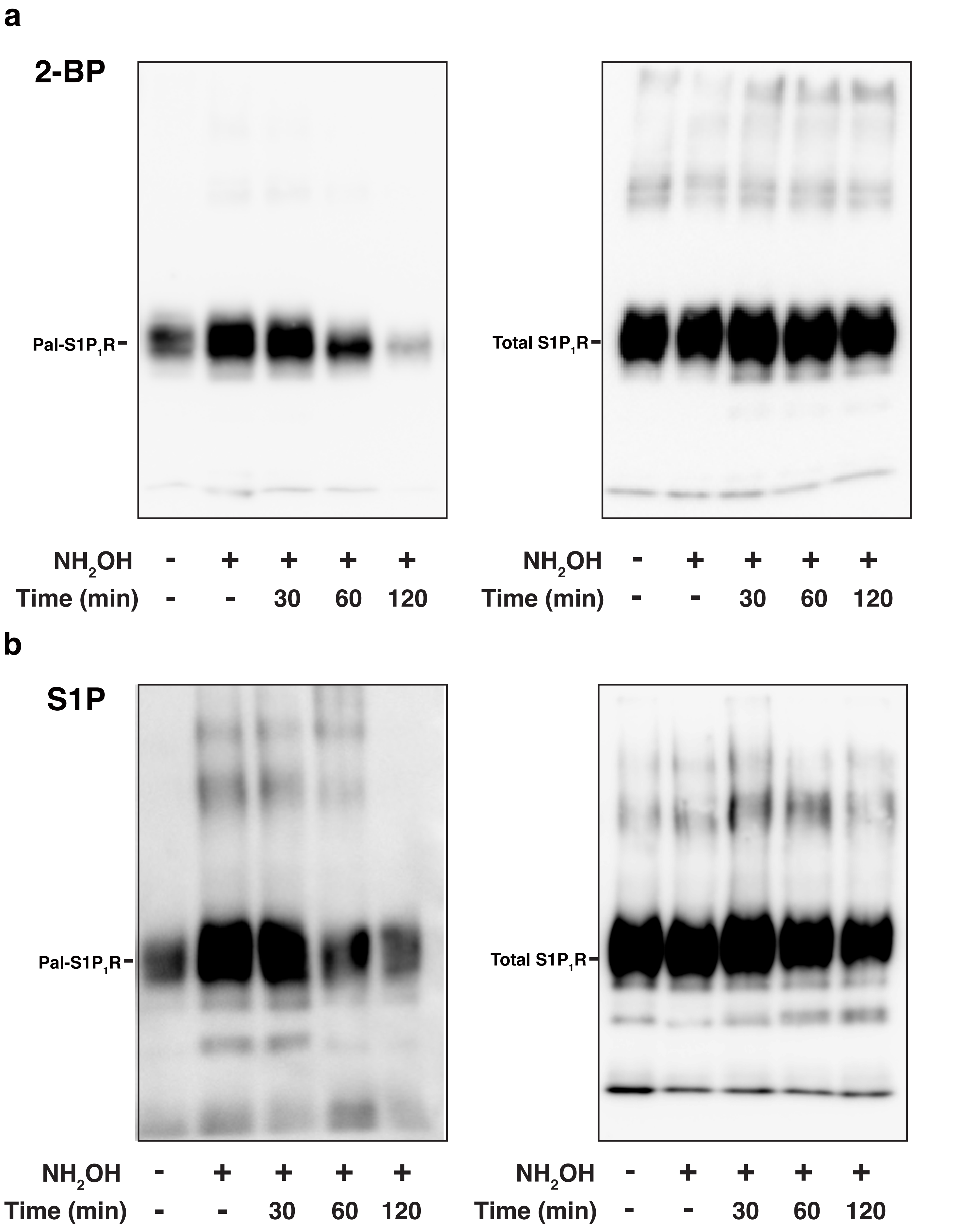

Supplementary Figure 8. Full-length image of western blot analysis in Figure 4.

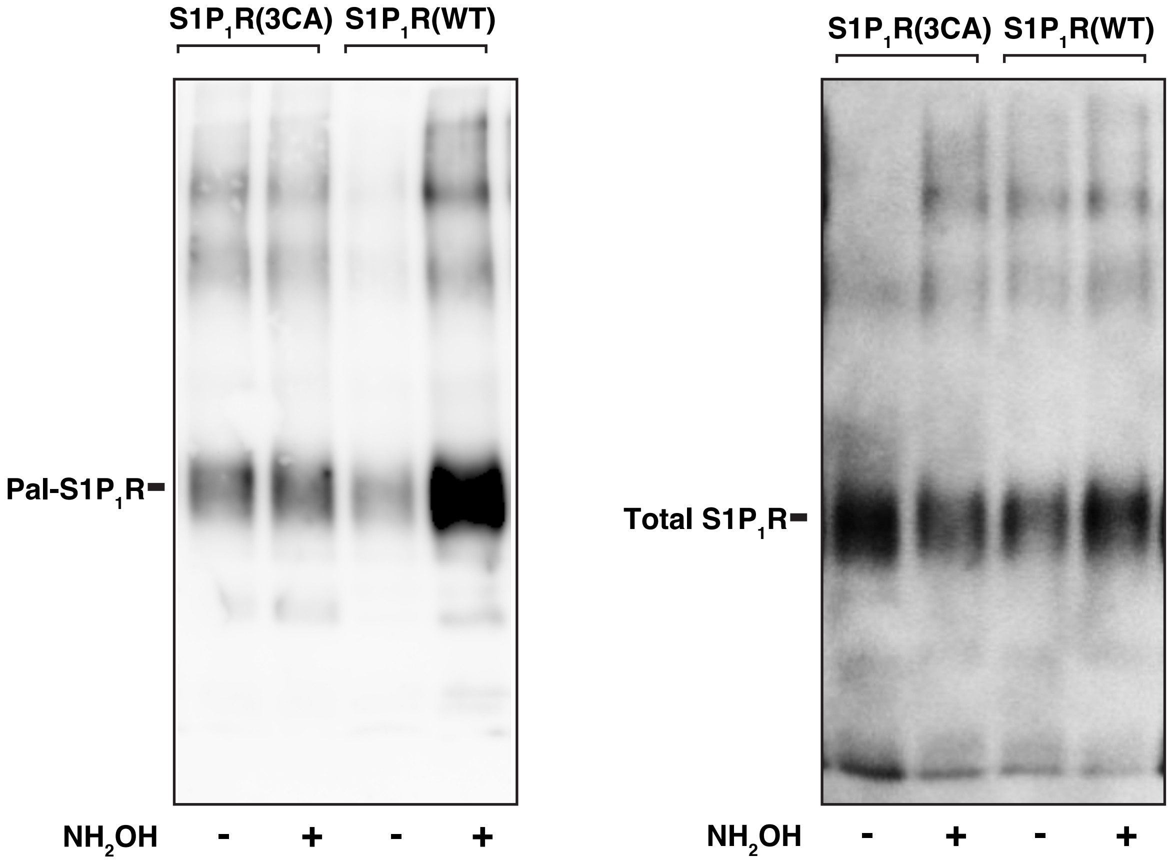

Supplementary Figure 9. Full-length image of western blot analysis in Figure 5.
